# Plant root tortuosity: an indicator of root path formation in soil with different composition and density

**DOI:** 10.1093/aob/mcw057

**Published:** 2016-05-03

**Authors:** Liyana Popova, Dagmar van Dusschoten, Kerstin A. Nagel, Fabio Fiorani, Barbara Mazzolai

**Affiliations:** ^1^Center for Micro-BioRobotics, Istituto Italiano di Tecnologia, Viale Rinaldo Piaggio 34, 56025 Pontedera, Italy; ^2^Institute of Bio- and Geosciences, IBG-2: Plant Sciences, Forschungszentrum Jülich GmbH, 52425 Jülich, Germany

**Keywords:** *Zea mays*, soil mechanical impedance, soil composition, soil density, root elongation, root path formation, gravity, MRI, root tortuosity

## Abstract

**Background and Aims** Root soil penetration and path optimization are fundamental for root development in soil. We describe the influence of soil strength on root elongation rate and diameter, response to gravity, and root-structure tortuosity, estimated by average curvature of primary maize roots.

**Methods** Soils with different densities (1·5, 1·6, 1·7 g cm^−3^), particle sizes (sandy loam; coarse sand mixed with sandy loam) and layering (monolayer, bilayer) were used. In total, five treatments were performed: Mix_low with mixed sand low density (three pots, 12 plants), Mix_medium - mixed sand medium density (three pots, 12 plants), Mix_high - mixed sand high density (three pots, ten plants), Loam_low sandy loam soil low density (four pots, 16 plants), and Bilayer with top layer of sandy loam and bottom layer mixed sand both of low density (four pots, 16 plants). We used non-invasive three-dimensional magnetic resonance imaging to quantify effects of these treatments.

**Key Results** Roots grew more slowly [root growth rate (mm h^–1^); decreased 50 %] with increased diameters [root diameter (mm); increased 15 %] in denser soils (1·7 vs. 1·5 g cm^–3^). Root response to gravity decreased 23 % with increased soil compaction, and tortuosity increased 10 % in mixed sand. Response to gravity increased 39 % and tortuosity decreased 3 % in sandy loam. After crossing a bilayered–soil interface, roots grew more slowly, similar to roots grown in soil with a bulk density of 1·64 g cm^–3^, whereas the actual experimental density was 1·48±0·02 g cm^–3^. Elongation rate and tortuosity were higher in Mix_low than in Loam_low.

**Conclusions** The present study increases our existing knowledge of the influence of physical soil properties on root growth and presents new assays for studying root growth dynamics in non-transparent media. We found that root tortuosity is indicative of root path selection, because it could result from both mechanical deflection and active root growth in response to touch stimulation and mechanical impedance.

## INTRODUCTION

Plant roots represent a good evolutionary example of successful soil penetration and exploration strategies. Roots are able to colonize the soil volume because they can efficiently penetrate the substrate and perform key functions for growth and survival, such as anchoring and foraging for nutrients and water (Gilroy and Masson, 2008). Optimal path selection by roots in the soil is fundamental for resource acquisition. The growing root consumes energy by respiring photoassimilates to find water and nutrients in an efficient way while avoiding hard substrates or nutrient-depleted environments. When facing a difficult environment, such as a hard-to-penetrate or dry soil layer, a single root may change the direction of growth, continue growing more slowly, or stop growing altogether. Root apex movements, such as tropic bending and circumnutations, play a significant role in root path selection during soil penetration. For example, by performing these movements, root apices may direct their growth through cracks or generally follow paths with a low impedance to penetration (Darwin and Darwin, 1897; Hart, 1990; Brown, 1993; Migliaccio and Piconese, 2001; Blancaflor and Masson, 2003; Gilroy and Masson, 2008; Migliaccio *et al.*, 2013).

The overall apex bending movement is a combination of both active bending and passive deflection. Studies of soil penetration have mostly addressed mechanical root deflection and buckling (Dexter and Hewitt, 1978; Whiteley and Dexter, 1984). Active bending, however, has mostly been considered in terms of obstacle circumnavigation (Massa and Gilroy, 2003*a*, *b*; Gilroy and Masson, 2008; Monshausen *et al.*, 2009; Toyota and Gilroy, 2013) or tropic responses to touch stimulation (Ishikawa and Evans, 1992; Gilroy and Masson, 2008). Although we can evaluate the overall bending response by analysing the waviness of the growth pattern, known as tortuosity (Inoue *et al.*, 1999; Buer *et al.*, 2000; Migliaccio and Piconese, 2001), quantitative estimates of the contribution of active growth responses relative to passive deflection during root bending in the soil remain lacking. Root tortuosity is generally studied to evaluate root anchorage (Dupuy *et al.*, 2005; Schwarz *et al.*, 2010*a*, *b*; Bengough *et al.*, 2011) and has also been related to the efficiency of seedlings establishment (Inoue *et al.*, 1999). However, root tortuosity may be the result of specific growth responses that depend on substrate characteristics and may enhance soil penetration and, indirectly, foraging efficiency. The elongation rate of the root apex is determined by both the root apex growth pressure and by the reaction force of the soil to its deformation (Greacen and Oh, 1972; Clark *et al.*, 2003). The endogenous physiological parameters determining the root apex growth pressure are osmotic potential, extensibility of the cell wall and wall tension (Schopfer, 2006). External environmental conditions that affect root elongation rate include the mechanical and bio-physical characteristics of the soil, such as water and nutrient concentration, soil compaction and composition, the presence of layers, and temperature (Bengough and Mullins, 1991; Bengough *et al.*, 1997, 2006, 2011; Buer *et al.*, 2000; Jin *et al.*, 2013).

Some of these factors are interconnected, which makes it difficult to separate effects of individual soil properties on root growth. For example, the mechanical strength of soil may increase with drying and thereby restrict root elongation (Whalley *et al.*, 2006). Bengough *et al.* (2011) examined the impedance to penetration with respect to water content in 19 soils with textures ranging from loamy sand to silty clay loam. At a matriz potential of −10 kPa, nearly 10 % of the measured penetration resistances were higher than 2 MPa, whereas at a matrix potential of − 200 kPa, the percentage rose to nearly 50 % (Bengough *et al.*, 2011), showing that soils with lower water content exhibited greater impedance to penetration.

Root growth can be arrested completely when the external pressure on the root apex exceeds the pressure that the root itself can exert (Goss, 1977; Clark *et al.*, 2003). If the external pressure on the root is high but still less than the root growth pressure, the root elongation rate generally decreases while diameter increases (Bengough and Mullins, 1990; Materechera *et al.*, 1992; Clark *et al.*, 2003; Bengough *et al.*, 2011; Jin *et al.*, 2013). From a mechanical viewpoint, the increased diameter may in turn lead to the development of a greater penetration force (Clark *et al.*, 2003), as well as to decreased buckling stresses (Bengough *et al.*, 1997), both of which enhance root penetration processes.

Although several studies addressed how soil features influence root growth, as summarized above, a quantitative estimation of these effects on growth parameters such as penetration rate, changes in root axis diameter and gravitropic response has seldom or only partially been performed due to the objective difficulties of accessing locally short-term (minutes to hours) root dynamics below ground. A significant step forward in this field would be to perform experimental studies in soils and dynamically extract data using a 4-D framework (3-D space and time). Moreover, to evaluate buckling and variations in growth, which are the most important parameters in evaluating root–soil interactions, measurements must be performed non-invasively. The primary obstacle to these kinds of studies relates to the non-transparency of soil and the need to investigate root movements dynamically. For these purposes, there are currently only a few suitable technologies, such as nuclear magnetic resonance imaging (MRI), X-ray and neutron computed tomography (CT) and positron emission tomography (PET) (Fiorani *et al.*, 2012; Metzner *et al.*, 2015). These techniques were not extensively available for 3-D root imaging 20 years ago, when most of the studies on the impact of soil mechanics on root growth were performed. Consequently, the use of these techniques can strongly contribute to increasing the existing knowledge on root–soil interactions and effects of soil-enforced bending and buckling on root mechanics as well as on tropic root growth responses.

To our knowledge, the present work is the first to describe the use of a 3-D imaging technique, MRI, to dynamically and non-invasively investigate how physical soil characteristics such as compaction, texture (i.e. relative composition based on particle-size distribution), and presence of layers with different properties influence the growth of primary maize roots in a 3-D environment. We evaluated root elongation rate and diameter, as well as the path selection described by root depth, response to gravity and tortuosity. We paid particular attention to assessing root tortuosity because this feature has, to our knowledge, never before been considered as a growth-related parameter in roots. We assume that tortuosity may vary with respect to the considered soil mechanical properties (i.e. composition and density) and believe that quantifying tortuosity may provide new insights into root path formation, because it reflects quantitatively bending processes during growth.

## MATERIALS AND METHODS

### Soil preparation

PVC cylinders with an 8-cm internal diameter (9-cm external diameter) and 30 cm in height were used as pots for MRI scanning. All strongly ferromagnetic particles were removed from the soil using permanent magnets after sieving. The pots were filled with mixed substrate, named here mixed sand (67 % coarse sand Ø1·4 mm on average, and 33 % Kaldenkirchen sandy loam composed of 73 % fine sand, 23 % coarse silt and 4 % clay) or sandy loam (Kaldenkirchen sandy loam composed of 73 % fine sand, 23 % loam and 4 % clay), each with a defined soil compaction. Compaction is directly proportional to soil bulk density. The soil in each pot was compacted as homogeneously as possible. A homogeneous packing was ensured by pouring in slightly wetted soil (up to 3 %, homogeneously wetted) and shaking afterwards (Supplementary Data, Fig. S1). The desired compaction level was achieved by filling the pot at once and carefully shaking the pot until the desired soil mass in a given volume was obtained. We used five types of experimental groups. The experiments with the first three groups, denoted Groups ‘Mix_low’/‘Mix_medium’/‘Mix_high’, were performed with mixed sand that was compacted at three different compaction levels in order to estimate the influence of soil density on the growth of primary maize roots. Experiments in a fourth group, denoted ‘Loam_low’, were performed using the sandy loam soil described above, which possessed a finer texture than the mixed sand, to evaluate how texture influences root growth. The fifth set of experiments, denoted Group ‘Bilayer’, was performed using a bilayer soil configuration: the top layer consisted of sandy loam and the bottom layer contained mixed sand as in Groups Mix. The density values of the soils in different experiments were approx. 1·5, 1·6 and 1·7 g cm^−3^, or low, medium and high, respectively (Table 1). We used three pots for Mix_low (12 plants), three for Mix_medium (12 plants), three for Mix_high (ten plants), four for Loam (16 plants) and four for Bilayer (16 plants).

**Table 1.** Soil bulk density and composition for each experimental group

	Bulk density (g l^−1^)	Type of soil	Soil physical characteristics
Group Mix_low	1·52±0·02	Mixed sand: 67 % coarse sand, 33 % sandy loam	low compaction coarse texture bulk structure
Group Mix_medium	1·59±0·02	Mixed sand: 67 % coarse sand, 33 % sandy loam	medium compaction coarse texture bulk structure
Group Mix_high	1·68±0·01	Mixed sand: 67 % coarse sand, 33 % sandy loam	high compaction coarse texture bulk structure
Group Loam_low	1·52±0·02	Sandy loam: 73 % fine sand, 23 % loam, and 4 % clay	low compaction fine texture bulk structure
Group Bilayer			
Bilayer_loam_top	1·27±0·04	Sandy loam layer: 73 % fine sand, 23 % loam, and 4 % clay	low compaction fine texture bilayer structure
Bilayer_mix_bottom	1·48±0·02	mixed sand layer: 67 % coarse sand, 33 % sandy loam	low compaction coarse texture bilayer structure

Abbreviations: Mix, mixed sand; Loam, sandy loam; Bilayer, bilayer soil structure.

Water content may modify the mechanical impedance of soil because it affects the interaction forces between soil particles. Therefore, the total volumetric water content was kept constant as much as possible for all plants during the experiment (approx. 15 % volumetric water content).

### Experiments and data acquisition

Experiments were performed using *Zea mays* (var. ‘Kubrick’, by Società Italiana Sementi S.p.A) seeds using a batch with a weight range of 0·2–0·3 g per seed. We began the experimental procedure by germinating the seeds on filter paper in a climate chamber (16/8 h day/night length with 20 °C day, 16 °C night, and a constant relative air humidity of 60±3 %) in the dark. We sowed 3-d-old maize seedlings (with a primary root length of approx. 2–3 cm) into the pots. Four seedlings were positioned equidistantly in each pot (about 3–4 cm from each other, 2 cm from the pot wall and 1 cm deep). We covered the soil surface with 1–2 cm of perlite after sowing to minimize water evaporation. Measurements started 1 d after sowing, after the plants had adapted to the new environment. Each experiment lasted 6 d. The pots were kept in the climate chamber at all times except during each MRI measurement session. Plants were grown under the same climate conditions as for germination. Lighting was provided by five 400-W HQI lamps (Philips, Hamburg, Germany) or five 400-W SON-T lamps (Philips) alternating every 2 h with a 5-min overlap, giving photosynthetically active radiation (PAR) of between 350 and 450 μmol m^−2^ s^−1^ at canopy level.

MRI scanning was performed daily using an MRI spectrometer with a 4·7-T, 30-cm vertical bore magnet and magnetic field gradients up to 300 mT m^−1^ (Varian, Palo Alto, CA, USA; Jahnke *et al.*, 2009). The spectrometer can generate 3-D volumetric data of water (^1^H imaging). Here, for discrimination between soil water and root water, the loamy sand particles caused a significant increase of the signal loss of soil water relative to that of root water (Schulz *et al.*, 2013). Under such conditions, root water is observed almost exclusively once the echo time is set appropriately (optimized for a high contrast-to-noise ratio). All experiments were performed using a spin echo multi-slice (SEMS) (Vlaardingerbroek and den Boer, 1999) sequence with an echo time (TE) of 9 ms and a repetition time (TR) of 3 s, during which time 100 slices were measured in parallel. The field of view (FOV) was 96 × 96 × 100 mm with a resolution of 0·5 × 0·5 × 1 mm^3^. Each image was acquired twice and averaged, resulting in a measurement time per slice block of 19·5 min. To form a complete root image, three slice blocks were acquired in about 1 h, and slice blocks were concatenated automatically using custom-made IDL (Interactive Data Language, ENVI software, Exelis Visual Information Solutions, Inc., Boulder, CO, USA), Python and C ++ scripts. By using a pick-and-place robot (Geiger, Aachen, Germany), we could fully automate plant positioning and measurements in the magnet.

Plants were harvested at the end of the experiment. Roots were washed out of the soil column and kept in 50 % ethanol before root diameter was analysed using WinRhizo 2012 software (Regent Instruments Inc., Québec, QC, Canada; settings: grey value threshold 30; removal of objects with an area <1 cm^2^ and a length/width ratio <4). Shoot length was measured from the seed to the shoot tip (the length of longest leaf) after harvesting. These measurements were performed to check if the treatments affected shoot development.

Mechanical resistance was quantified using a hand-penetrometer (hand-penetrometer for top layers, type IB, Eijkelkamp, Giesbeek, the Netherlands). This penetrometer was slowly inserted (about 2 cm s^−1^) manually in the centre of the pot before harvesting the plants. The values of spring compression (cm) with respect to depth were annotated and then transformed into MPa according to the procedure provided by the manufacturer. To find the penetration pressure (expressed in N cm^−2^), the annotated spring compression (cm) was multiplied by a factor of 2 [this value is associated with the spring used (5 N) and a cone of cross-section area 0·25 cm^2^] and divided by 100 to obtain MPa. The mean values of the soil mechanical impedance at 8-cm depth were chosen for comparison: Group Mix_low, 0·02 MPa; Group Mix_medium, 0·04 MPa; Group Mix_high, 0·25 MPa; and Group Loam_low, 0·22 MPa.

### Data analysis and evaluation

After the initial image reconstruction, the images were converted into graphs using software described by Schulz *et al.* (2013). Briefly, visible structures with a tube-like appearance in the 3-D MRI images were connected to form a ‘tree’ or wireframe, also referred to as wireframe data. The lengths of individual roots were automatically extracted from these wireframes. The root elongation rate was calculated for each root as the difference of the root tip positions between two measurement time points divided by the elapsed time between the two measurements.

**Fig. 1.**
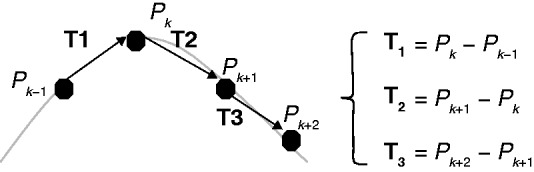
Graphical representation of points, P, and vectors, T, to calculate the SOAM index. Vectors, T, are constructed from points, P, which describe the root structure. Adapted from Bullitt *et al.* (2003).

For analysis of root diameter, a 1-cm-long root region 1 cm behind the root tip was considered for all experiments. This region was chosen because roots generally show an increase in diameter in the mature region that also produces secondary roots. In primary maize roots, the mature region starts 2–5 cm behind the root tip, depending on soil characteristics, such as hardness and moisture (Bengough and Mullins, 1990; Bengough *et al.*, 2006, 2011). It was not possible to measure the root diameter in the top layer of Group Bilayer (Group Bilayer_loam_top) because the diameter was evaluated only at the end of the experiment when the primary root tip had already grown through the bottom layer (Group Bilayer_mix_bottom). To evaluate changes in rooting depth, we calculated the ratio between root depth and length, referred to as depth-vs.-total length. Depth was calculated as the vertical distance (mm) between the initial (first measurement point) and final positions of the root tip. The total primary root length was calculated from the wireframe data of the last experimental day, i.e. the sixth day. This parameter can also be used to evaluate the gravitropic response by quantifying the amount of downward growth with respect to length growth. This ratio depends on both the root tortuosity and the tangent of growth direction. The ratio between horizontal growth and vertical growth, termed the horizontal-vs.-vertical growth ratio, also quantifies the root response to gravity because it can be seen as the tangent of the gravitropic set-point angle. Vertical and horizontal growth were calculated from the wireframe data on the sixth experimental day by summing the vertical and horizontal projections between two consecutive pixels along the entire primary root. The ratios were calculated by dividing the averaged horizontal elongation rate by the averaged vertical elongation rate. Here, tortuosity effects are excluded (differently from the depth-vs.-total length parameter). Different types of tortuosity can be calculated for a curve (Bullitt *et al.*, 2003). For example, one of the simplest methods is to calculate how far a path deviates from a straight line by dividing the curve length by the linear distance between the endpoints. This metric may assign the same value to both a single, large curve and multiple small ones. To overcome this limit, additional multiplication by the number of turns can be performed. Different approaches exist to identify the number of turns, such as calculating the number of inflection points or intersections with the midline. However, coils (as in a spring) do not contain inflection points and do not intersect their midline. Thus, to overcome this challenge and calculate the tortuosity of 3-D root structures, an approach based on estimating the curvature was chosen. Root tortuosity was calculated using the sum of angles metric (SOAM) index described by Bullitt *et al.* (2003). The SOAM index is calculated as the curvature integrated along a curve and normalized by the path length (Bullitt *et al.*, 2003). A positive total angle of curvature (or in the case of discrete points, an equivalent angle between two given vectors in space) at point *P_k_* (Fig. 1) is calculated by taking the square root of the sum of the squares of the in-plane angle and of the torsional angle. The total angles are summarized for each valid point in the curve, and the result is normalized by dividing by the total curve length. Results are given in rad mm^−1^ in the present study. The in-plane angle at point *P_k_* (*IP_k_*) and the torsional angle *TP_k_* are given by the following equations, where *TP_k_*, *IP_k_* ϵ [0, π]:

(1) $${IP}_{k}=\cos^{-1}\left(\left(\frac{T_{1}}{\left|T_{1}\right|}\right)\cdot \left(\frac{T_{2}}{\left|T_{2}\right|}\right)\right)$$

(2) $${TP}_{k}=\cos^{-1}\left(\left(\frac{T_{1}\times T_{2}}{\left|T_{1}\times T_{2}\right|}\right)\cdot \left(\frac{T_{2}\times T_{3}}{\left|T_{2}\times T_{3}\right|}\right)\right)$$

The total angle *CP_k_* at point *P_k_* is then

(3) $${CP}_{k}=\sqrt{\left({IP}_{k}\times {IP}_{k}\right)+\left({TP}_{k}\times {TP}_{k}\right)}$$

The SOAM calculates the total tortuosity of the curve as

(4) $$SOAM=\;\frac{\left(\sum_{k=1}^{n-3}{CP}_{k}\right)}{\left(\sum_{k=1}^{n-1}\left|P_{k}-P_{k-1}\right|\right)}$$

### Statistical analysis

Table 2 summarizes the number of plants considered in the experiments and the number of measurements for the different root growth characteristics. Primary roots in two plants of Group Mix_high did not develop at all after planting. Root wireframes data could not always be estimated when roots crossed each other, due to the difficulty in linking correctly and automatically the root wireframes of different measurement points (once in Group Mix_low and once in Group Mix_medium). In this case, the tortuosity and horizontal-vs.-vertical growth ratio were estimated using only that part of the root that could be correctly analysed (i.e. five partial roots were considered in Group Mix_low and one in Group Mix_medium) or could not be analysed at all (two roots in Group Bilayer_mix_bottom). However, the absolute length of each wireframe that was analysed should not significantly influence the tortuosity index (Bullitt *et al.*, 2003) or the horizontal-vs.-vertical growth ratio. The root elongation rate on day 6 (calculated as difference between measurement points on day 5 and day 6 divided by the elapsed time) was not estimated for roots that had reached the bottom of the pot.

**Table 2.** Number of measurements for each primary root trait

	Group Mix_low	Group Mix_medium	Group Mix_high	Group Loam_low	Group Bilayer_loam_top	Group Bilayer_mix_bottom
No. of plants	12	12	10	16	16	16
Root elongation rate	47	59	50	79	16	44
Root diameter	12	12	10	16	nd	16
Horizontal-vs.-vertical growth ratio	12	12	10	16	14	16
Tortuosity	12	12	10	16	14	16
Final shoot length	12	12	10	16	nd	16

Values indicate the number of measurements for each group; nd, not determined.

All the correlations were calculated using the Pearson coefficient (Miller *et al.*, 1965) and based on averaged values of each pot (i.e. the average was calculated from plants belonging to the same container).

## RESULTS

### The effect of soil density and texture on root growth dynamics

**Fig. 2.**
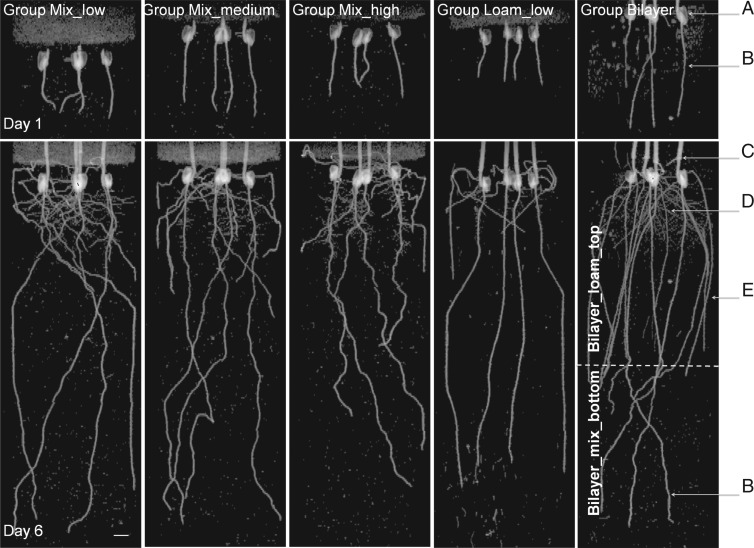
Typical MRI projection images of maize roots growing in mixed sands with different bulk densities (Groups Mix), textures (mixed sand of Group Mix_low and sandy loam of Group Loam_low with the same bulk densities) and structures (Group Bilayer with layered soil structure). The images are 2-D projections of 3-D data along the *z–x *plane (where *z* is the vertical axis, *x* and *y* are perpendicular planar axes). Four plants per pot can be seen, and their organs are indicated: (a) seed, (b) primary root, (c) shoot, (d) secondary and (e) seminal roots. The plants are 4 and 9 d old at Day 1 and Day 6 of the experiment, respectively. The perlite covering used to limit water evaporation is visible as a defuse layer on the top. The grey spots represent remaining soil water as well as some small weed plants that accidentally germinated in the soil during the experiments. The dashed line on the image of Group Bilayer represents the interface between the upper sandy loam layer, Group Bilayer_loam_top and the lower mixed sand layer, Group Bilayer_mix_bottom. The white bar represents 10 mm.

**Fig. 3.**
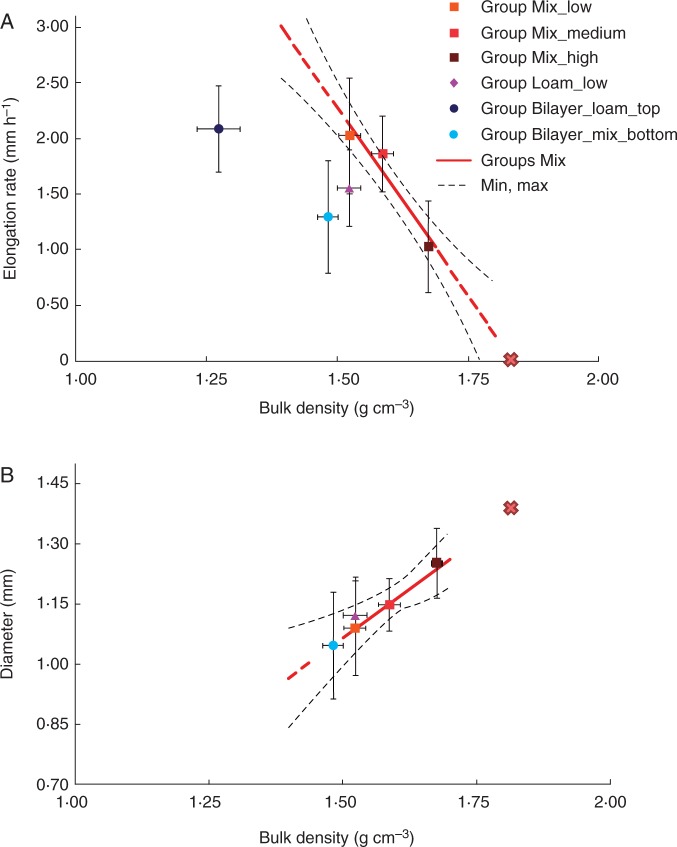
Root elongation rate (A) and diameter (B) with respect to soil bulk density, texture and soil structure for primary maize roots. Mean values are shown ±s.d. for each group. Solid red lines represent the linear regression model for Groups Mix (see Methods for mathematical formula), whereas the dashed red lines show the extrapolated values that are out of the data fitting region, and the dashed black lines indicate the confidence limits of the model prediction (90 % confidence). The red X indicates the estimated bulk density and root diameter for root growth arrest according to the linear models of root growth responses in the mixed sand of Groups Mix.

In Fig. 2, we show typical MRI images obtained on the first and the last day of MRI experiments for all five groups, shown as 2-D projections of the 3-D MRI data. The primary roots clearly grew less in more compacted soils (i.e. roots in Group Mix_low grew deeper than roots in Groups Mix_medium and Mix_high) and increased their diameter with increasing soil compaction (Fig. 3). The calculated Pearson correlation between increased soil bulk density and decreased root elongation rate was very high, *r*=0·91 (*P* < 0·001, *t*-test), and the linear regression model was found to be statistically significant. The Pearson correlation between changes in the soil bulk density and root diameter was *r*=0·76 (*P* < 0·05, *t*-test). Thus, both root elongation rate and root diameter are correlated in an opposite way with soil bulk density.

The roots also appear more tortuous in the mixed sand of Group Mix_high than in the sandy loam of Group Loam_low. Similarly, segments of the same root were more tortuous in the mixed sand layer than in the sandy loam layer in Group Bilayer (Fig. 2). In addition, the plants in Group Bilayer presented seminal roots that were considerably longer than in other groups, specifically Groups Mix_low and Loam_low. The observed root diameters of the plants in the Bilayer_mix_bottom layer had similar conditions to the Group Mix_low (Table 1), in accordance with predictions made from the linear regression of Groups Mix (the predicted and experimental values were the same: 1·05 vs. 1·05±0·13 mm, respectively). Interestingly, the root diameter of Group Loam_low was also similar to that of Group Mix_low (i.e. within the confidence limits of the linear regression model) the two groups having the same soil compaction level, whereas the elongation rate of roots grown in Group Loam_low was lower than those in Group Mix_low (Fig. 3). These results indicate that root diameter, unlike root elongation rate, depends only on soil bulk density and does not appear to depend on soil texture or the presence of layers. The daily elongation rate of the primary roots in Groups Mix and Loam did not vary significantly (Supplementary Data, Fig. S2). The treatments probably did not significantly impact shoot growth (Supplementary Data, Fig. S3), probably because maize development is dependent on seed reservoirs during the first 2 weeks after germination (Hochholdinger and Tuberosa, 2009).

Finally, rooting depth is important for plant anchorage and for acquiring water that is possibly present and often more abundant in deeper sub-soil layers. The observed changes in rooting depth may depend on different growth parameters, such as elongation rate and path selection. We concentrated on studying how soil physical characteristics, in particular compaction, texture and structure, influence different growth parameters.

### Characterization of root path selection with respect to soil density and texture

Primary roots generally tend to grow downward because of gravitropism. Interactions between gravitropism and other tropic responses may lead to decreased rooting depth as well as decreased vertical growth with respect to horizontal growth. We used root depth with respect to total root length as well as the ratio between horizontal and vertical root growth to determine the influence of soil mechanical impedance on gravitropism. For instance, higher depth-vs.-total length and lower horizontal-vs.-vertical growth ratios indicate stronger root responses to gravity. Here, the gravitropic response is defined as the inverse of the horizontal-vs.-vertical growth ratio. Mathematically, the depth-vs.-total length ratio cannot be greater than 1. In our experiments, this parameter ranged between 0·7 and 0·9 with comparatively greater values indicating deeper growing roots. The horizontal-vs.-vertical growth ratio may assume any positive value; values less than 1 indicate that vertical growth is greater than horizontal growth and vice versa. In all groups, the horizontal-vs.-vertical growth ratio was lower than 1, ranging from 0·25 to 0·75, meaning that vertical growth was dominant. When root growth is totally downward, the depth-vs.-total length ratio equals 1, while the horizontal-vs.-vertical growth equals zero. We never observed exclusively vertical growth in our experiments; there was always a component of horizontal growth as well.

**Fig. 4.**
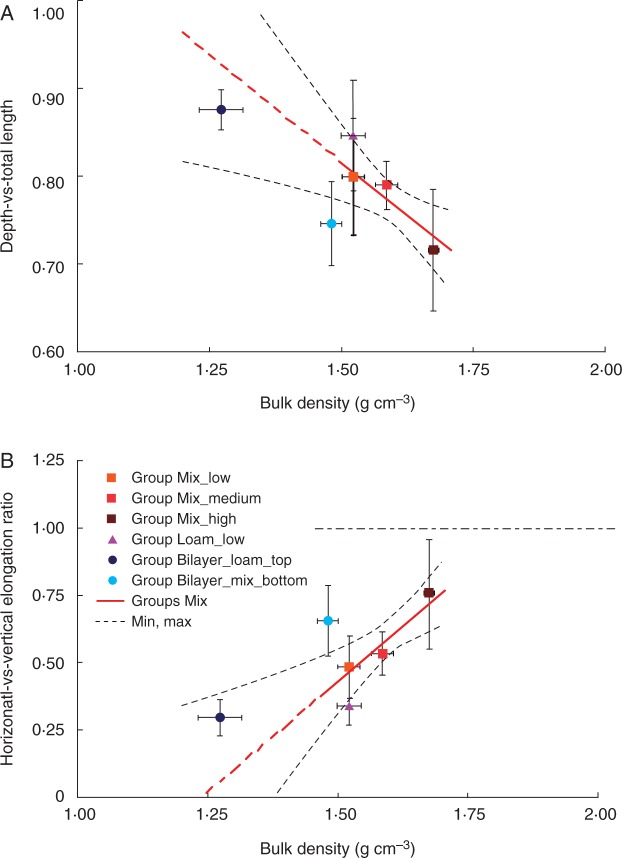
Depth-vs.-total length (A) and horizontal-vs.-vertical growth (B) of roots with respect to soil bulk density, texture and soil structure for primary maize roots. Mean values are shown ±s.d. for each group. Solid red lines represent the fitting linear regression model for Groups Mix (see Methods for the mathematical formula), whereas the dashed red lines show the extrapolated values out of the data fitting region, and the dashed black lines indicate the confidence limits of the model prediction (90 % confidence). The dashed horizontal black line in B divides the plot into two regions: a lower region in which vertical growth is greater than horizontal growth and an upper region in which horizontal growth is greater than vertical growth.

**Fig. 5.**
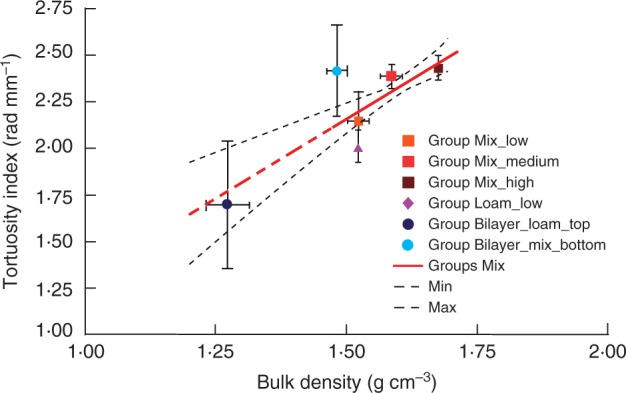
Root tortuosity with respect to soil density. Mean values are shown ±s.d. for each group. The solid red line represents the fitting linear regression model for Groups Mix (see Methods for the mathematical formula), whereas the dashed red lines show the extrapolated values out of the data fitting region, and the dashed black lines indicate the confidence limits of the model prediction (90 % confidence).

The depth-vs.-total length ratio was up to 11 % lower, and the horizontal-vs.-vertical growth ratio was approx. 30 % higher for roots grown in the soil with higher compaction, Group Mix_high, compared with roots grown in looser soil, Group Mix_low (Fig. 4). These differences indicate that the ability of roots to follow gravity becomes less pronounced in the higher bulk density of the mixed sand (Fig. 4). These two parameters, depth-vs.-total length and horizontal-vs.-vertical growth ratio, were found to be highly correlated in our experiments, with a Pearson coefficient of *r*=–0·99 calculated for all groups (*P* < 0·0001), indicating that the vertical growth component was exclusively downward (not upward). The horizontal-vs.-vertical growth ratio was correlated with root elongation rate. The Pearson coefficient was *r* = –0·88 for roots grown in Groups Mix (*P* < 0·01), meaning that the decreased elongation rate correlates with a decrease in the gravitropic response. We found a decrease in elongation rate due to the increased mechanical impedance of the soil; thus, soil mechanical impedance influences path selection by interacting with and/or dominating the gravitropic response in Group Loam_low, where the mechanical impedance was lower than in Group Mix_high but higher than in Group Mix_medium, where the horizontal-vs.-vertical growth ratio was lower than in the mixed sand of Groups Mix. In other words, root growth aligned more with the gravity vector of roots grown in sandy loam than for those grown in mixed sand, indicating that path selection by primary roots is also influenced by soil texture. Roots in Groups Mix exhibited a different level of tortuosity than roots in Group Loam_low. The roots were straighter in sandy loam, whereas they were more tortuous in mixed sand (Fig. 2). The average tortuosity index (calculated as SOAM) was higher for roots grown in soil with a higher compaction and lower for roots grown in sandy loam than for the mixed sand (Fig. 5). The tortuosity of roots grown in Group Bilayer_loam_top was lower than for those grown in Group Sandy_low, probably due to the lower bulk density, as in the Groups Mix. These trends represent the first attempt to provide some additional information on how roots respond to the soil environment. The tortuosity index showed a weak negative correlation with the elongation rate, i.e. the Pearson coefficient was *r* = –0·56 considering all groups. Tortuosity was highly correlated with the depth-vs.-total length and horizontal-vs.-vertical growth ratios, i.e. the Pearson coefficients were *r* = –0·85 and *r* = 0·80 considering all groups (*P* < 0·001 from the *t*-test), indicating that increased tortuosity is correlated with a decrease in the downward growth component in our experiments.

### Linear statistical models of growth parameters with respect to the soil bulk density and statistical significance

We performed linear regression analysis of the data from Groups Mix to obtain the relationship between soil bulk density and growth parameters, represented by the solid red lines in Figs 3–5. We evaluated the significance of data trends with respect to the soil bulk densities in Groups Mix. The significance of the Pearson correlation coefficient between the dependent variable and the soil bulk density was evaluated using *t*-tests, while the differences between constant and linear trends of the dependent variable with respect to the soil bulk density were subjected to F-tests (Table 3). Constant models (i.e. mathematical relationship between two parameters expressed by a constant function) were built from average values. The data were considered to be significant at *P* < 0·05. Pearson correlations indicate linear relationships between elongation rate, diameter, depth-vs.-total length and horizontal-vs.-vertical growth ratio with respect to soil bulk density, with the linearity of the relationships being significant for all models from the F-test.

**Table 3.** Pearson correlation and F-test values between constant and linear regression models† of dependent variables with respect to soil bulk density along with their statistical significance

Dependent variable	Pearson correlation	Constant model	Linear model	F-test to compare constant and linear models
Elongation rate	–0·91 ***	*y* = 1·62	*y* = –6·77*x* + 12·43	26·09 **
			*R*^2^ = 0·66	
Diameter	0·76 *	*y* = 1·17	*y* = 0·99*x* – 0·42	17·08 **
			*R*^2^ = 0·59	
Depth-vs.-total length	–0·81 **	*y* = 0·77	*y* = –0·51*x* + 1·59	11·62 **
			*R*^2^ = 0·49	
Horizontal-vs.-vertical elongation ratio	0·82 **	*y* = 0·59	*y* = 1·64*x* – 2·03	16·62 **
			*R*^2^ = 0·58	
Tortuosity	0·64 ·	*y* = 2·32	*y* = 1·70*x* – 0·39	11·97 *
			*R*^2^ = 0·42	

† *x* is soil bulk density and *y* is the dependent variable.

Significant correlation and differences between constant and linear models:

***P < 0·001, **P < 0·01, *P < 0·05, and ·*P* < 0·1.

### Analysis of root elongation rate and path selection with respect to soil layering

The growth parameters of Group Bilayer_mix_bottom were quite different from those of Group Mix_low, even though in both cases we grew the roots in mixed sand with a low bulk density. Indeed, the elongation rate, depth-vs.-total length ratio and tortuosity of roots of Group Bilayer_mix_bottom were greater than expected based on the results of Groups Mix, whereas the vertical-vs.-horizontal growth ratio was lower than expected (Fig. 3A, 4 and 5). Only the root diameter of Group Bilayer_mix_bottom was consistent with the results of Groups Mix (Fig. 3B).

## DISCUSSION

### Influence of soil density and texture on growth dynamics of primary roots

Increasing the soil compaction resulted in a reduced root elongation rate and an increased root diameter, with elongation rate and diameter showing a negative correlation. These results are in line with published data (Taylor, 1980; Bengough and Mullins, 1990; Materechera *et al.*, 1992; Misra and Gibbons, 1996; Clark *et al.*, 2003; Bengough *et al.*, 2011; Jin *et al.*, 2013). The numerical data reported in the above mentioned literature vary significantly between different species. For example, our findings on the root elongation rate and diameter of Group Mix_high are very similar to those reported by Bengough and Mullins (1990) although our data also compare the root growth responses for different soil densities, compositions and structures.

Our findings suggest that the elongation rate depends on the resistance of the soil to penetration, which is in agreement with previous findings showing that the root elongation rate is sensitive to variations in axial pressure (Bengough and MacKenzie, 1994; Bengough, 2012) but insensitive to radial pressure (Kolb *et al.*, 2012). Interestingly, we found that changes in root diameter probably depend on the soil bulk density but do not significantly depend on the soil texture.

The adjustment between root elongation rate and root penetration force depends strongly on the mechanical interactions between the penetrating root and the soil. In the case that the penetration pressure remains the same and the root diameter increases, the root penetration force should increase as a function of the increasing cross-sectional area (i.e. the square of the root diameter). By contrast, Tonazzini *et al.* (2012, 2013) observed that the penetration force of an artificial probe increased linearly with the radius increasing during penetration tests into granular substrates. As a consequence, the penetration pressure acting on the probe was much lower than expected for probes with a larger radius, because the penetration force depended on the probe radius rather than on its cross-sectional area. A similar condition may occur in growing roots, where the increased diameter would allow the root to penetrate substrates with higher penetration resistance at the same root penetration pressure. These results may explain the positive linear relationship between root diameter and soil bulk density (Farrell and Greacen, 1966; Goodman and Ennos, 1999; Jin *et al.*, 2013), where soil bulk density can be considered an indicator of the soil penetration resistance.

Changes in root elongation rate and diameter are generally associated with increased mechanical impedance (Panayiotopoulos *et al.*, 1994; Clark *et al.*, 2003; Bengough *et al.*, 2011; Jin *et al.*, 2013). Spring barley also shows dynamic compensatory root and shoot growth responses when exposed to localized soil compaction and fertilization applied in rhizoboxes (Pfeifer *et al.*, 2014). Thus, by extrapolating the root growth response from the soil physical characteristics, it is also possible to estimate the maximum limit of soil compaction to prevent root arrest. For instance, the linear regression model extrapolates that root growth arrest could occur at a soil density of 1·84 g cm^−3^ with a root diameter of 1·40 mm (Fig. 3). These values are reasonable, as maize root growth arrest was reported for a penetration resistance of 0·8–2 MPa (Clark *et al.*, 2003; Bengough *et al.*, 2011), which corresponds to a soil bulk density of 1·7–2 g cm^−3^ depending on the soil texture (Whalley *et al.*, 2007). In our experiments, two plants did not grow (approx. 17 % of plants in the group) at the highest used density, i.e. Group Mix_high, probably due to the high compaction (penetration resistance 0·25 MPa). The different elongation rates observed for roots grown in soils with the same compaction but with different textures (i.e. Group Mix_low and Group Loam_low) may have different causes: (1) the mechanical impedance of sandy loam may be higher compared with mixed sand of the same bulk density (Kirby and Bengough, 2002); and (2) the oxygen content of sandy loam is lower than that of mixed sand (Moldrup *et al.*, 2001; Horn and Smucker, 2005). Although we evaluated the mechanical impedance of the soil using a manual penetrometer, and thus the measurements are qualitative rather than quantitative, the results show that the resistance to penetration in Group Loam_low soil was higher than in Group Mix_low soil and was comparable with Group Mix_high soil [average penetrometer resistance of 0·25±0·08 MPa (*n* = 3) and 0·22±0·06 MPa (*n* = 4) for Group Mix_high and Loam_low, respectively, at 8 cm depth]. On the seventh day in undisturbed soil, we also registered a decreased oxygen content, which was probably due to bacterial activity (data not shown). Thus, the decreased elongation rate of roots in Group Loam_low compared with Group Mix_low was probably due to the increased mechanical impedance of the soil.

### Influence of soil density and texture on the gravitropic response

The gravitropic response of roots is important for plant anchorage and orientation in soil to position themselves within available soil nutrients. Gravitropism is often partially inhibited by other tropisms, such as light, temperature and touch (Gilroy and Masson, 2008). The interplay between the responses to gravity and touch may allow roots to identify paths with lower mechanical impedance. Thus, a reduction of the gravitropic responses in roots may indicate some alterations in root path selection induced by other stimuli.

In this study, in addition to the canonical parameter that describes root depth with respect to root total length, we also used the ratio between horizontal and vertical root elongation rates as an indicator of the gravitropic response. These two parameters describe a similar phenomenon and may be highly correlated. However, they provide different information. For example, if a root grows horizontally in a spiral pattern, its depth-vs.-total length would be approximately zero, whereas its horizontal-vs.-vertical growth ratio would depend on the spiral shape and not be related to the depth-vs.-total length ratio. Therefore, we suggest that tracking these two parameters during root growth may help to explain which and how soil conditions influence root growth. We found that the root response to gravity decreases with higher soil compaction (Fig. 3), which probably grants roots a higher degree of freedom in changing their growth direction and thus increasing the probability to follow paths with lower mechanical impedance. We found that soil texture also influences the gravitropic response. In particular, the primary roots grew more downward in soil with a finer texture. In the soil of Groups Mix, there is an abundance of large sandy particles embedded in the soil matrix. If a root tip cannot shift such particles, it must circumnavigate the particle, with a consequent increase in tortuosity and horizontal movement. For finer soil texture, the likelihood of encountering an unmovable particle is significantly less, thus allowing a gravitropic response to dominate over the obstacle circumnavigation response or mechanically enforced deflection. To our knowledge, the gravitropic root response to soil texture is a novel finding, and should encourage additional experiments with different soil textures to better quantify and understand this phenomenon.

### Tortuosity – an indicator of root path formation

Tortuosity is a result of root bending and changing growth direction; therefore, we can consider it as an indicator of the path formation process. It may reflect, at least to some extent, the tropic action towards an optimal path for growing in response to counterbalancing inputs, such as gravitropism and local soil impedance, as well as the tropic growth for water, nutrients and oxygen. Assuming that root growth is not random but is given by passive bending and a thigmotropic response (Ishikawa and Evans, 1992; Gilroy and Masson, 2008), following a path with less impedance is probably more ecologically convenient than simply growing straight down. Calculating the root tortuosity index is a way to estimate changes in root growth direction. The SOAM tortuosity index, adopted here (Bullitt *et al.*, 2003), is particularly useful for comparing average curvature without taking into account the distance from the midline. This metric is mathematically different from tortuosity, calculated by dividing a straight path by total length. Thus, this method provides different information than depth-vs.-total length and the horizontal-vs.-vertical growth ratio and does not correlate mathematically with these parameters; any eventual correlation found experimentally depends on experiment and data processing. The high correlation registered in our experiments shows that higher tortuosity indices corresponded to decreased gravitropism, which makes sense given that a root would need to perform consecutive bendings and thus overcome the gravitropism in order to form a tortuous structure.

The SOAM tortuosity index gives higher values for tighter coils with equal total lengths. It provides information regarding how rapidly root bending occurs during overall growth and thus the prevalence of differential elongation with respect to symmetrical elongation. If differential elongation is the result of a particular response to external or internal stimuli, such as tropic responses or endogenous nutations, then it can vary under several conditions, and thus its quantification may be useful for comparing root growth in different environments.

Our results show that the tortuosity of primary roots is indicative of the different soil bulk densities and textures. We observed in Group Bilayer that tortuosity of roots increased by 38 % after they passed the interface between two layers. Similarly, the tortuosity of Groups Mix was also higher than that of Group Loam. The differences in tortuosity could be due to differences in soil texture between sandy loam and mixed sand. Coarse sand particles in the mixed sand left cavities on the root border, indicating localized pressure (Supplementary Data, Fig. S4). This pressure may lead to root bending driven by touch stimulation (Ishikawa and Evans, 1992) or to the mechanically enforced bending, which can in turn be some of the causes of tortuosity formation.

**Fig. 6.**
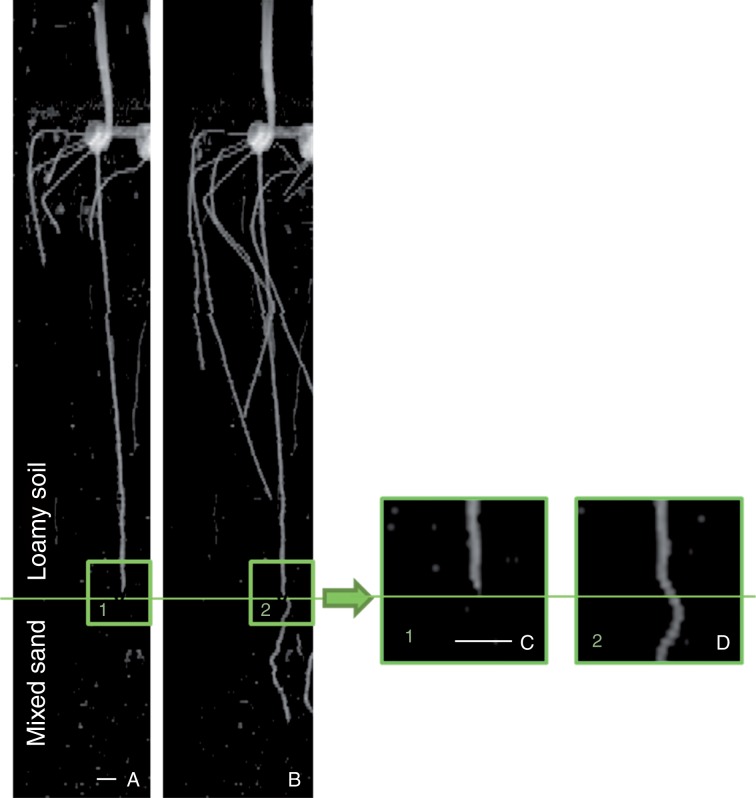
Typical 2-D projection MRI images of primary root grown in loamy soil (A) before entering the mixed sand and (B) after penetrating the mixed sand layer. Higher magnification (3×) images show a root that is (C) straight prior to entering the bottom layer and (D) curved just at the top of the mixed-soil layer. The images are the 2-D projections of 3-D data along the *z–x* plane. Scale bar, 10 mm.

In some cases, we observed that roots buckled upon encountering the second layer (Fig. 6). To better observe the buckling phenomenon, we performed a dedicated experiment with primary maize roots grown in mixed sand (soil bulk density, 1·34 g cm^−3^) containing a round, flat, glass obstacle (8 cm in diameter) positioned horizontally at a depth of 8 cm. In this experiment, after coming into contact with the obstacle, the root buckled markedly some millimetres over the root bending due to the obstacle (see time-lapse video in Supplementary Data; time-lapse MRI scanning was performed every 20 min). Similar buckling or passive bending may also occur at the interface between the two layers of the bilayer soil, with the lower layer being harder to penetrate than the upper layer, or when the root apex encounters a soil aggregate or particle that it cannot penetrate or displace (Whiteley and Dexter, 1984).

In summary, on the basis of our observations, we hypothesize that root tortuosity could result from two processes: (1) passive, soil-enforced mechanical bending and/or buckling, which we define here as passive bending; and (2) active bending in response to touch stimulation induced by soil inhomogeneity. We do not know yet which phenomenon, passive or active, is prevalent. However, both may depend on the likelihood of the root apex encountering a hard soil aggregate or sand particle. The inhomogeneity of mixed sand may induce active bending due to touch stimulation (Ishikawa and Evans, 1992; Gilroy and Masson, 2008) along with passive bending due to difficulty in penetrating and/or displacing the larger soil particles (Whiteley and Dexter, 1984).

Root tortuosity plays a role in plant anchorage and in local root stabilization to prevent buckling and uplifting during penetration by apex growth (Inoue *et al.*, 1999; Dupuy *et al.*, 2005; Schwarz *et al.*, 2010*a*, *b*; Bengough *et al.*, 2011). Increased tortuosity may increase the root’s capacity to penetrate soil. However, the growth of a tortuous structure may require more energy and resource allocation compared with straight roots. There should be some trade-off between the two requirements of penetrating the soil and consuming less energy or fewer resources. Thus, changes in root tortuosity could serve as an indicator of changing growth responses due to adaptations to the micro-environment.

### Root growth adjustments in bilayered soil structures

The type of soil environment, whether homogeneously structured or layered, was found to influence root growth from the perspective of the root apex. For example, the elongation rate was significantly reduced (approx. 30 %) for roots grown in layered soil compared with roots grown in un-layered soil of similar density (Fig. 3A).

We can use the linear regression models of growth parameters with respect to the soil bulk density for Groups Mix (described in Table 3, column 4) to extrapolate the growth parameters for the other given soil densities (Table 4, column 3) as well as to calculate an equivalent soil bulk density for given experimental growth parameters (Table 4, column 4). In the case of Group Bilayer_mix_bottom, the averaged experimental values of all the growth parameters considered, except root diameter, correspond to an equivalent soil bulk density of 1·63–1·65 g cm^−3^ (Table 4, column 4, Figs 3–5). For example, the elongation rate and gravity response of the primary roots in Group Bilayer_mix_bottom matched those calculated for the equivalent soil bulk density of approx. 1·64 g cm^−3^ (Table 4, column 5), even though the experimental bulk density was much lower (1·48 g cm^−3^). Thus, the bilayered soil structure that we used here appears to exert a considerable influence on root growth behaviour. These effects are important to consider, particularly when evaluating the growth response of roots growing in natural soils, which are generally layered and not homogeneous, as well as in experiments where different layers of soil and/or artificial substrates are adopted.

**Table 4.** Prediction of the primary root growth parameters for Group Bilayer_mix_bottom and comparison with the experimental data

Growth parameter	Group Bilayer_mix_bottom exp. growth parameters (1·48±0·02 g cm^−3^)	Predicted growth parameters for a soil bulk density of 1·48 g cm^−3^	Equivalent soil bulk density for the exp. growth parameters	Growth parameters for an equivalent soil bulk density of 1·64±0·01 g cm^−3^
Elongation rate	1·29±0·51	2·41	1·63	1·33
Depth-vs.-total length	0·75±0·05	0·84	1·65	0·75
Horizontal-vs.-vertical elongation ratio	0·66±0·13	0·40	1·64	0·66
Tortuosity	2·42±0·24	2·13	1·65	2·42

The linear regression models are presented in Table 3. Predicted growth parameters are calculated by putting the soil density of Group Bilayer_mix_bottom (1·48 g cm^−3^) into the linear models based on the linear regression of Groups Mix (Table 3, column 4). Equivalent soil bulk density was calculated by putting the growth parameters of Group Bilayer_mix_bottom into the reversed aforementioned linear models. The averaged estimated soil bulk density was then put into the same linear models (Table 3, column 4) to calculate the predicted growth parameters. The growth parameters for an equivalent soil density are similar to the measured growth parameters and are within the standard deviation of measured values.

In addition to the marked decrease of primary root growth, we observed an increased growth of seminal roots in the Group Bilayer_mix_bottom with respect to the growth of seminal roots in other groups (Supplementary Data, Fig. S5). This root growth adjustment may be one of the mechanisms of root adaptation to changing environments that somehow results in higher levels of plant development. For example, the decrease of elongation rate in roots that reached the hardpan may free up carbon resources to promote the growth of other roots in softer soil layers. The described growth changes in primary root of Group Bilayer could be due to the abrupt changes in soil compaction. These changes are in agreement with the theory postulated by Toyota and Gilroy (2013), who hypothesized that roots could display either phasic or tonic reactions to gravitropic and mechanical stimuli depending on whether the stimulus was transient or continuous. Indeed, the compacted soil of Groups Mix and Loam can be seen as continuous mechanical stress, whereas the interface between two soil layers of different bulk density and texture in Group Bilayer represents a transient stress on the root apices. Toyota and Gilroy (2013) reported higher values of response signal for transient stresses than for tonic stresses of the same magnitude. This could explain the significant decrease in the root elongation rate and gravity response obtained in the bilayered soil structure relative to the predictions (Fig. 4). The possible existence of two kinds of response, phasic and tonic, would probably provide some advantage to roots that are exploring the soil volume. For example, in the case of an abrupt increase in impedance (such as reaching a hardpan, similar to the Group Bilayer in our experiment), the root may stop elongating even though the soil impedance does not exceed the root penetration pressure and promote the growth of other roots in more favourable micro-environments.

### Implications

Mechanical impedance and soil composition are major factors affecting root growth. Our results regarding the influence of soil compaction on root elongation rate and root diameter are in agreement with existing literature. However, our results point to a crucial role for soil texture and layering in causing significant differences in root growth. We propose the SOAM index as one of the parameters to be considered when studying root growth. By analysing the root gravitropic response and tortuosity, we found that soil density and texture has a quantitative influence on root path formation.

This type of investigation under varying conditions of soil compaction, texture and structure was enabled by the application of cutting-edge plant phenotyping technology, specifically MRI, which allows investigators to observe root growth and development non-invasively in three dimensions by growing plants in soil. We strongly believe that non-invasive 3-D imaging is well suited for investigating both single root growth and root apparatus development with respect to a variety of soil characteristics, such as soil mechanics and composition, water potential, and nutrient and oxygen content. In particular, it is possible to use MRI to evaluate variations in root growth responses to local differences in water content and nutrient spots. Here, we focused on the primary root although similar experiments can be performed with other root types, such as seminal and lateral, and with different genotypes. The plant root’s abilities to explore soil efficiently have also been recently considered as a source of inspiration for developing root-like robots (Mazzolai *et al.*, 2011; Sadeghi *et al.*, 2013, 2014). A better comprehension of how living roots have evolved to penetrate and move into the soil efficiently, as well as in-depth investigation into particular characteristics, such as adaptive growth, energy-efficient movements and the ability to penetrate soil at any angle, are of particular interest from an engineering perspective. The key features extrapolated from these studies can then be translated into specifications for developing bio-inspired robots that move and act in soil analogously to their natural counterparts for environmental monitoring and exploration tasks.

## SUPPLEMENTARY DATA

Supplementary data are available online at www.aob.oxfordjournals.org and consist of the following. Figure S1: typical MRI projection images showing the homogeneity of the water profile, and thus the associated homogeneity of the density profile. Figure S2: daily elongation rate of primary roots. Figure S3: length of the shoot (9-d-old plants). Figure S4: typical images of seminal root development in Groups Mix_low, Loam_low and Bilayer. Figure S5: longitudinal section of a maturation region of a primary root grown in: (A) sandy loam and (B) mixed sand.

Supplementary Data
