# Supplementary material for: Plant root tortuosity: an indicator of root path formation in soil with different composition and density
Source: Ann Bot. 2016 May 3;118(4):685–98. doi: 10.1093/aob/mcw057 (PMC5055621; doi:10.1093/aob/mcw057)
Supplement: Supplementary Data [file supp_118_4_685__index.html]

Plant root tortuosity: an indicator of root path formation in soil with different composition and density — Supplementary Data 

# Plant root tortuosity: an indicator of root path formation in soil with different composition and density

## Supplementary Data

files

- Supplementary Data - zip file
