# Supplementary material for: Plant root tortuosity: an indicator of root path formation in soil with different composition and density
Source: Ann Bot. 2016 May 3;118(4):685–98. doi: 10.1093/aob/mcw057 (PMC5055621; doi:10.1093/aob/mcw057)
Supplement: Supplementary Data [file supp_mcw057_suppl_data.zip › aob-15685-s01.docx]

**Figure supplementary material legends**

**Supplementary Fig. 1** Typical MRI projection images showing the homogeneity of water profile, and thus the associated homogeneity of density profile: (A) projection maps along 2 axis, and (B) maximum intensity projection maps. The maize plant was 3 weeks old and was grown in the mixed sand. A small artefacts due to MRI rf pulse in homogeneity are visible. Details of imaging: FOV=10*36 cm, resolution 3x3x3 mm^3^, 6 3D images of 6 cm each were used to build the total image, TE is 0.8ms, TR is 600ms. Images were taken half a day after watering.

**Supplementary Fig. 2** Daily elongation rate of primary roots. values are shown +/- SD for each group.

**Supplementary Fig.3**Length of the shoot (nine-day-old plants). Shoot length was measured as the distance between the seed and the leaf tip. Mean values are shown +/- SD for each group.

**Supplementary Fig.4**Typical images of seminal root development in Groups Mix_low, Loam_low, and Bilayer. Images are 2D projections of 3D data along the *zx* plane. Bar, 10 mm.

**Supplementary Fig.5** Longitudinal section of a maturation region of a primary root grown in: A) sandy loam, wherein the root is straight with regular cell walls, and B) mixed sand, wherein the root walls are irregular and present some cavities with strongly attached soil particles. Images were captured on a light microscope (Axiophot 2 Zeiss). Bar, 0.5 mm.
