# Supplementary figures and images for: Plant root tortuosity: an indicator of root path formation in soil with different composition and density
Source: Ann Bot. 2016 May 3;118(4):685–98. doi: 10.1093/aob/mcw057 (PMC5055621; doi:10.1093/aob/mcw057)

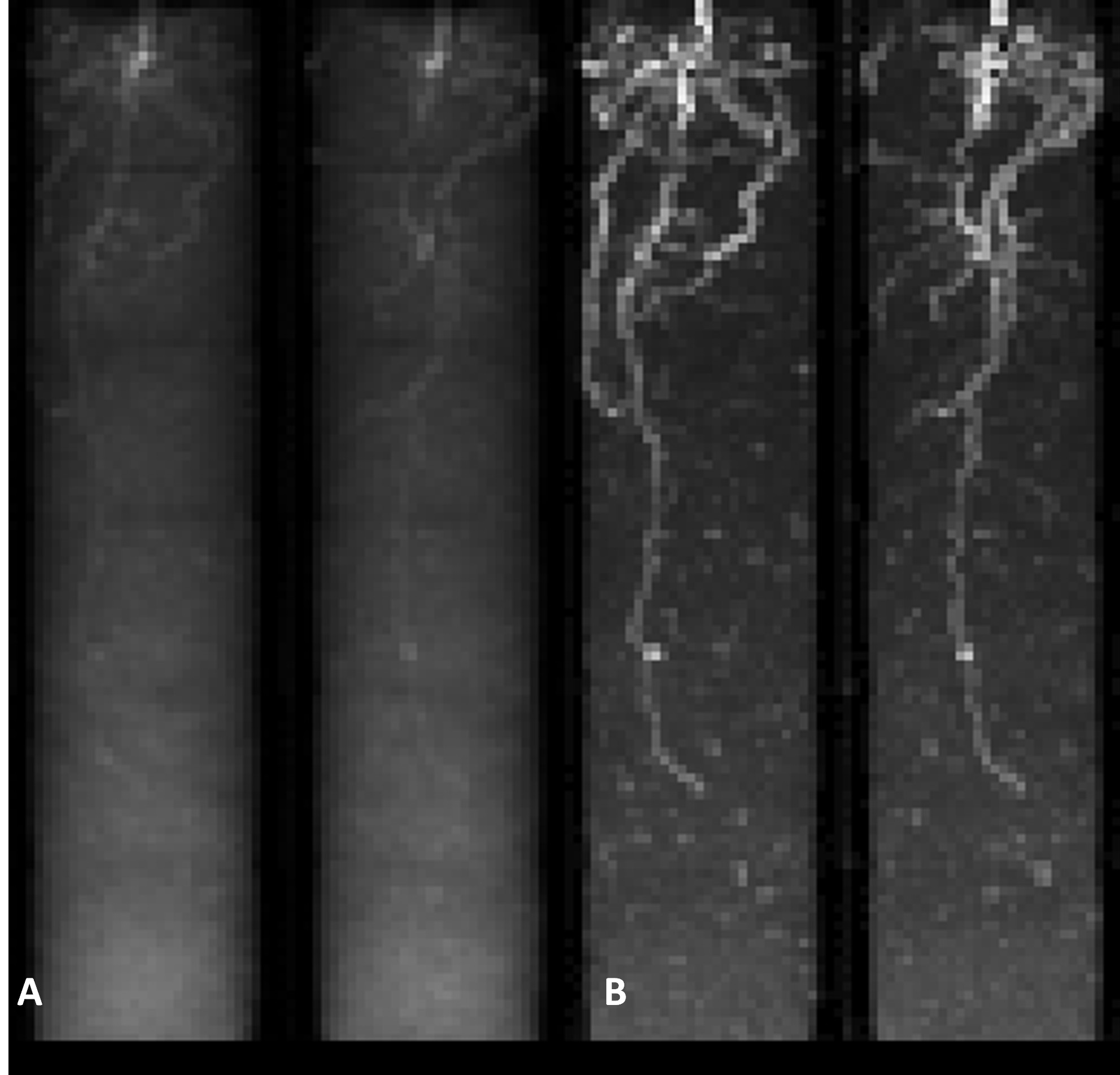

Supplement: Supplementary Data [file supp_mcw057_suppl_data.zip › aob-15685-s03.jpg]

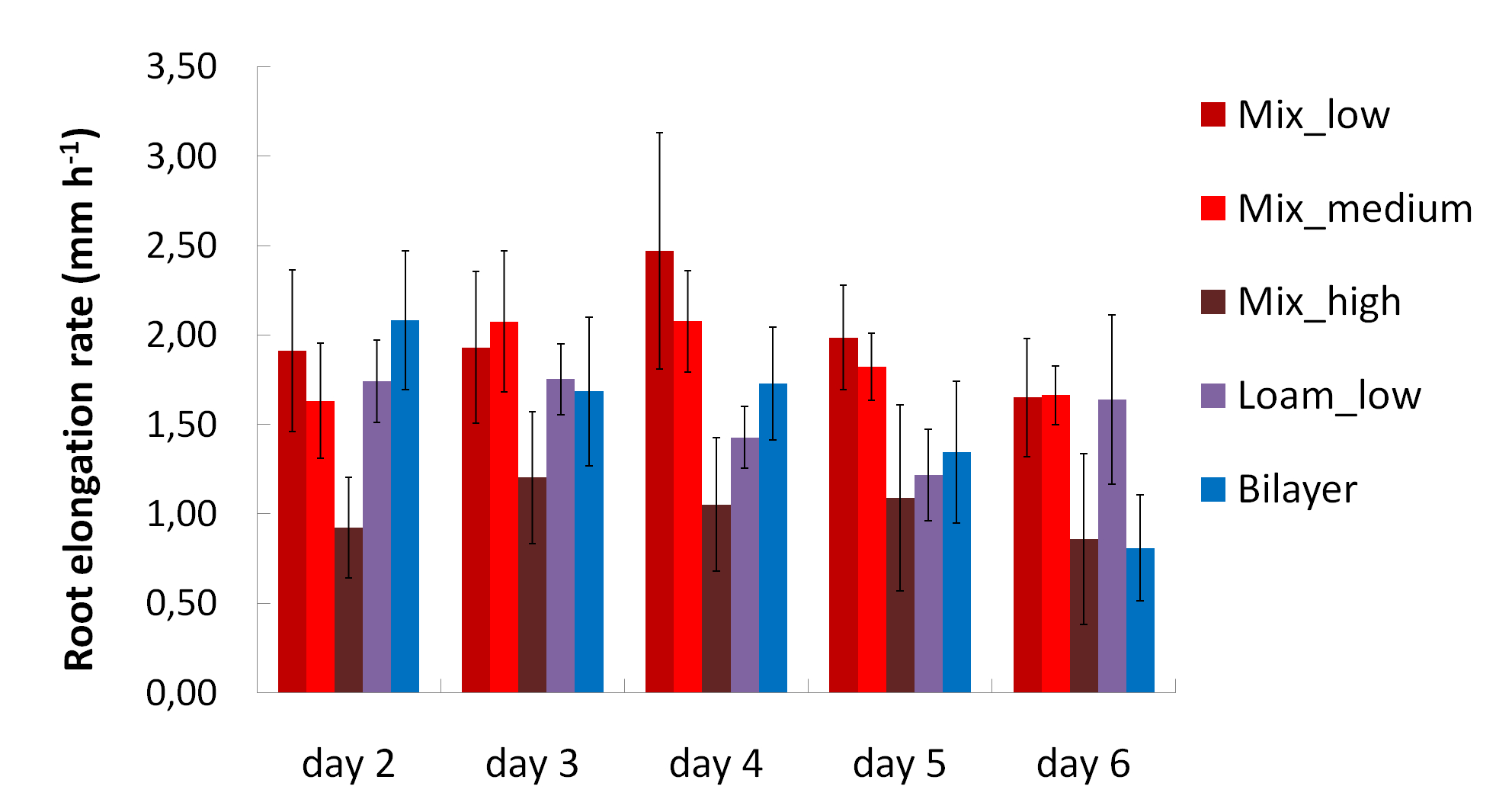

Supplement: Supplementary Data [file supp_mcw057_suppl_data.zip › aob-15685-s04.jpg]

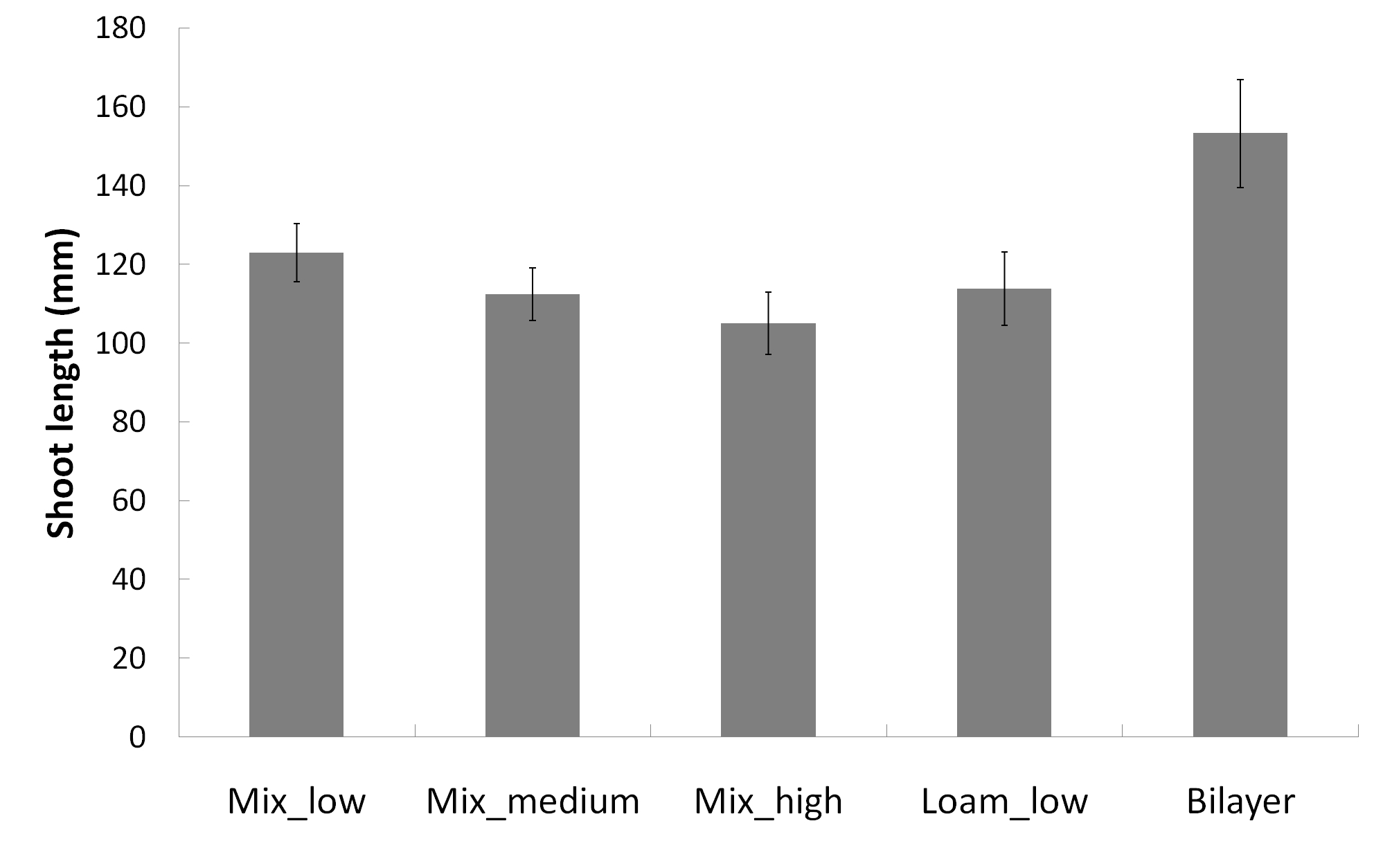

Supplement: Supplementary Data [file supp_mcw057_suppl_data.zip › aob-15685-s05.jpg]

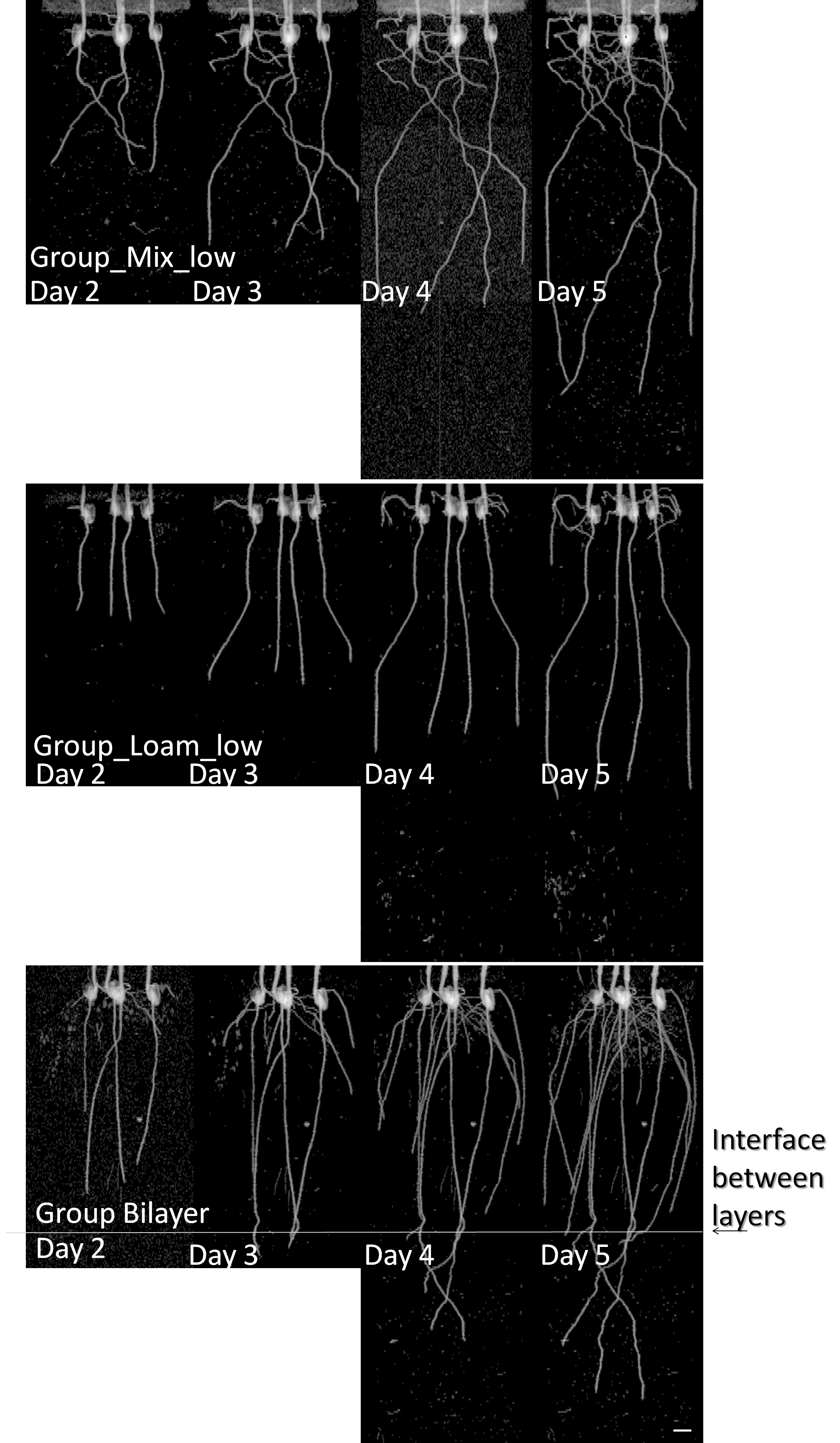

Supplement: Supplementary Data [file supp_mcw057_suppl_data.zip › aob-15685-s06.jpg]

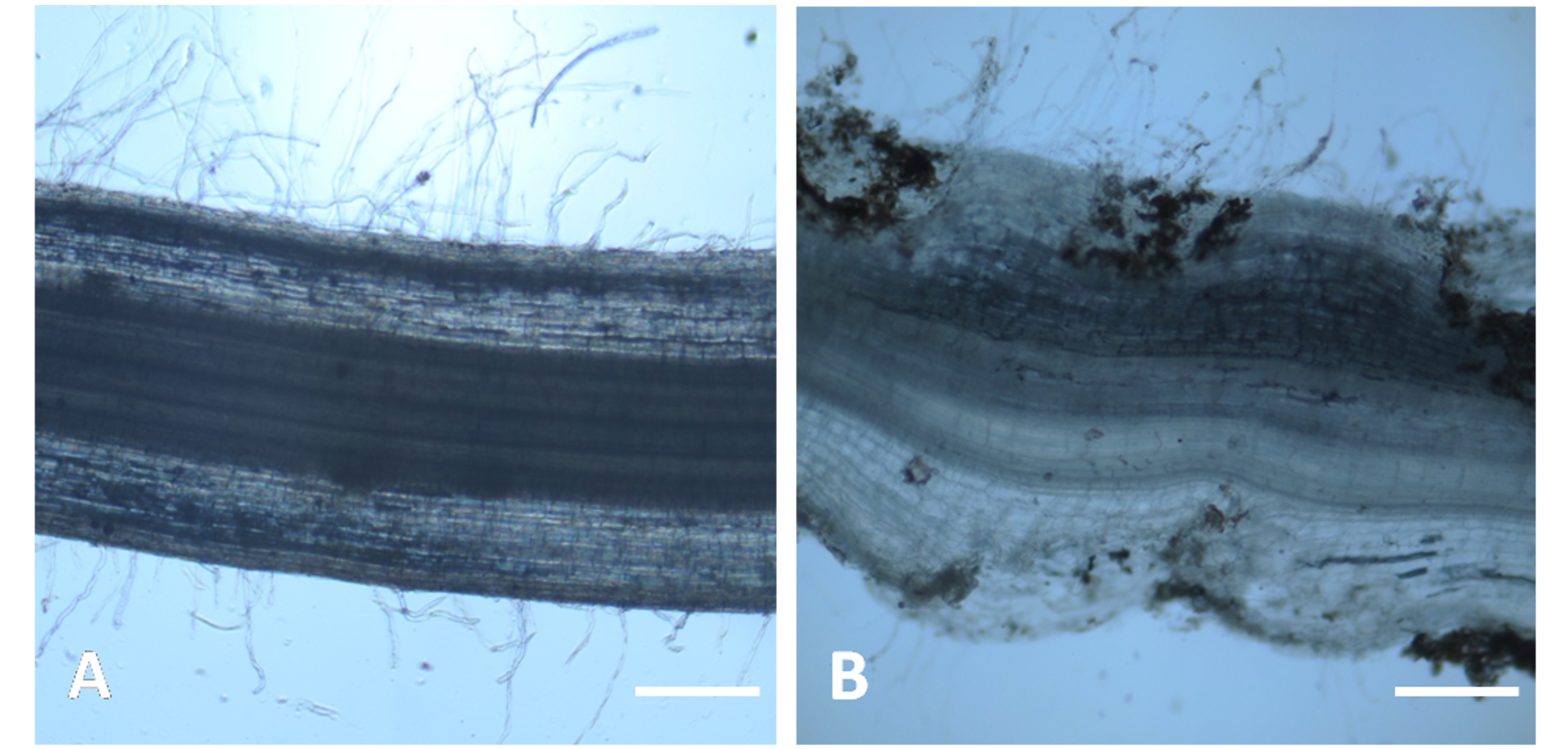

Supplement: Supplementary Data [file supp_mcw057_suppl_data.zip › aob-15685-s07.jpg]
